# Hyperacusis Assessment Questionnaire—A New Tool Assessing Hyperacusis in Subjects with Tinnitus

**DOI:** 10.3390/jcm12206622

**Published:** 2023-10-19

**Authors:** Danuta Raj-Koziak, Elżbieta Gos, Justyna Jolanta Kutyba, Piotr H. Skarzynski, Henryk Skarzynski

**Affiliations:** 1 Tinnitus Department, World Hearing Center, The Institute of Physiology and Pathology of Hearing, 02-042 Warsaw, Poland; d.koziak@ifps.org.pl; 2 Teleaudiology and Screening Department, World Hearing Center, The Institute of Physiology and Pathology of Hearing, 02-042 Warsaw, Poland; j.kutyba@ifps.org.pl (J.J.K.); p.skarzynski@ifps.org.pl (P.H.S.); 3 Institute of Sensory Organs, 05-830 Kajetany, Poland; 4 Heart Failure and Cardiac Rehabilitation Department, Faculty of Medicine, Medical University of Warsaw, 03-242 Warsaw, Poland; 5 Otorhinolaryngology Clinic, World Hearing Center, The Institute of Physiology and Pathology of Hearing, 02-042 Warsaw, Poland; skarzynski.henryk@ifps.org.pl

**Keywords:** hyperacusis, decreased sound tolerance, tinnitus, questionnaires

## Abstract

Hyperacusis, a kind of decreased sound tolerance, is difficult to measure objectively. It often co-occurs with tinnitus. There is a need for valid and reliable patient-reported outcome measures to capture this subjective phenomenon. The aim of the study was to create a questionnaire capturing hyperacusis in terms of loudness, fear, and pain and to evaluate its psychometric properties. The study sample consisted of 106 adult patients with hyperacusis and tinnitus with a mean age of 45.2 years. A medical interview, an audiological examination, and several questionnaires (the Tinnitus Handicap Inventory, the Hyperacusis Questionnaire, the State–Trait Anxiety Inventory, and Visual Analog Scales) were applied. The final 14-item Hyperacusis Assessment Questionnaire showed an appropriate three-factor structure with 70.5% of the variance explained. Convergent and divergent validity were confirmed by correlations with other measures of hyperacusis, anxiety, tinnitus severity, misophonia, and hearing thresholds. The internal consistency assessed with Cronbach’s alpha was excellent (α = 0.91), as was reproducibility (intraclass correlation, ICC = 0.96). The new Hyperacusis Assessment Questionnaire is a psychometrically sound and brief tool assessing the severity of hyperacusis in terms of loudness, fear, and pain. It can be used in clinical practice and scientific research for patients with hyperacusis and tinnitus.

## 1. Introduction

Hyperacusis is a challenge both for patients and clinicians because of its subjective nature and debilitating character. It negatively affects the quality of life, emotional well-being, hearing, sleep, and concentration and causes psychiatric disorders in some patients [1,2]. There are very few data on the prevalence figures for hyperacusis. Research conducted in 1999 showed hyperacusis in 15.3% of respondents [3]. Further studies showed hyperacusis occurrence in a range from 6 to 16% in the general population [4,5,6]. In children, 3.7% confirmed that they experienced oversensitivity or distress to particular sounds [7]. Based on recently published data in the general population, the prevalence of hyperacusis is 0.2–17.2%. In people with specific conditions, such as tinnitus, the prevalence is higher [8].

The field of hyperacusis is young, and a variety of terms and definitions have been proposed to describe it [9]. Based on a consensus that followed a standard Delphi methodology, hyperacusis has been defined as a reduced tolerance to sound(s) that are perceived as normal to most of the population or were perceived as normal to the person before their onset of hyperacusis where “normal” refers to sounds that are generally well tolerated [10]. In our opinion, the above definition contains not only a great deal of subjectivity (however, this must be the case because the phenomenon itself is subjective), but it is also overly general. It does not include any specific defining feature that can be grasped, e.g., during an interview with a patient. Our clinical practice shows that this key feature is the perceived loudness of external sounds. Patients complain that they suffer from sounds that are too loud for them. The umbrella term decreased sound tolerance, proposed by Jastreboff and Jastreboff [11], seems to be more appropriate to capture multiple types of sound tolerance disorders, while the term hyperacusis should be reserved for excessive loudness sensation.

Therefore, from our point of view, the most useful approach is that proposed by Tyler et al. [12], namely that hyperacusis should be categorized as loudness, annoyance, fear, or pain. Loudness Hyperacusis is considered to be a basic psychoacoustic response; it occurs when even moderately intense sounds are judged to be very loud. Annoyance hyperacusis is described as a negative emotional reaction to sounds, not always reported to be loud, and the reaction can be specific to a particular group of sounds. Hyperacusis fear is an aversive response to sounds resulting in avoidance behavior. The experience of pain after exposure to even moderately intense sounds is called Pain Hyperacusis [1]. However, we think that the perception of loudness is the predominant feature in hyperacusis and is accompanied by a reaction at the emotional level (fear) as well as a reaction at the physical level (pain). However, in our opinion, annoyance is not a feature of hyperacusis; this instead refers to misophonia.

Our position is, to some extent, consistent with the findings made by Fackrell et al. [13]. A qualitative analysis of the types of problems experienced by adults with hyperacusis showed that they could be categorized into three general categories: fear, reduced quality of life, and physical reaction to sound. Fear was very often reported by patients as problematic; they felt fearful, frightened, and worried in relation to sounds. The category “reduced quality of life” included problems with everyday functioning caused by hyperacusis. The category “physical reaction to sound” referred to physical pain and physical discomfort due to sounds. Therefore, the three categories that we have named following Tyler (loudness, fear, pain) are broadly in line with those identified by Fackrell et al. [13].

Hyperacusis is considered to be a subjective, self-reported symptom of some physiological change in the central auditory system [9]. The mechanism of hyperacusis is not completely understood. It has been proposed that it involves an aberrant increase in central auditory gain; however, there is a lack of a satisfactory animal model [14,15,16,17]. The central gain model remains the most empirically supported model of hyperacusis proposed to date and posits that the symptoms of hyperacusis are generated by a pathological increase in activity from central auditory structures, such as the inferior colliculus and the primary auditory cortex, at suprathreshold intensities [14,18]. Hyperactive responses in the primary and secondary auditory cortex are thought to encode the loudness rather than the intensity of a sound and are hypothesized to be responsible for Loudness Hyperacusis [19,20]. Sound-evoked fMRI activity in the human primary and secondary auditory cortex is increased in subjects with higher Hyperacusis Questionnaire scores [21].

There is no cure for hyperacusis [1]. However, some therapeutic procedures have been implemented and have centered upon the avoidance of provocative stimuli, desensitization by gradual sound exposure [22,23], acoustic training [24], the use of sound generators in Tinnitus Retraining Therapy [25,26], and Cognitive Behavioral Therapy. Minimally invasive surgery, involving reinforcing the oval and round windows and stapes superstructure, has been developed as a potential treatment for hyperacusis patients [27,28].

Hyperacusis and tinnitus may occur together, and Tyler and Conrad-Armes [12] were the first to show the relationship between them. Tinnitus is the perception of a sound without any external auditory stimulation, and its estimated prevalence is 10% to 15% in adults [29]. Jastreboff and Jastreboff [30] estimate that 60% of tinnitus patients have significantly decreased sound tolerance, and 30% of these suffer from hyperacusis and require treatment for it. Anari reported that 86% of adults with hyperacusis as the primary complaint experience tinnitus as well [31]. Hyperacusis is commonly observed among patients with chronic tinnitus, and those who experience both hyperacusis and tinnitus require more frequent management (intervention) than those who experience just one symptom [32]. The central gain model of hyperacusis assumes that peripheral hearing loss (as a consequence of reduced neural output from the cochlea—i.e., sensorineural hearing loss or cochlear synaptopathy) triggers compensatory changes in the central auditory system, which can give rise to tinnitus and hyperacusis [18]. The precursor of tinnitus and hyperacusis could also be comorbid. It has been found that teenagers who experienced transient tinnitus while in a sound-proof booth had loudness discomfort levels 10 dB lower than teens who did not experience tinnitus in the booth [33].

Both hyperacusis and tinnitus are subjective and so are measured by self-report questionnaires. Different tools are available to assess specific aspects of tinnitus, with the most frequently used ones being the Tinnitus Handicap Inventory [34,35] and the Tinnitus Functional Index [36]. However, there are only a few questionnaires available to assess hyperacusis. The most popular seems to be the Hyperacusis Questionnaire (HQ) developed by Khalfa et al. [37], but the independent validation of HQ done by Fackrell et al. [38] suggests that it does not provide a valid overall measure of hypersensitivity. The conclusions relate to the use of HQ when measuring hyperacusis in the tinnitus population. The Multiple-Activity Scale for Hyperacusis (MASH) is an instrument that considers 14 activities or places (e.g., concerts, shopping centers) where noise discomfort can be experienced and captures the level of annoyance on a scale from 1 to 10 [39]. Another tool, the Hyperacusis Handicap Questionnaire (HHQ), was developed for individuals with tinnitus associated with hyperacusis [40]. The authors used a clinical sample consisting of 50 patients, but descriptions of the tool’s development and evidence for its validity are scarce. A new and interesting tool is the Inventory of Hyperacusis Symptoms (IHS), created by Greenberg and Carlos [41] to measure hyperacusis in research and clinical practice. It was originally validated not in a clinical sample but in an online general population that self-reported auditory sensitivity. Clinical validation of the tool was conducted by Aazh et al. [42], who concluded that IHS has good internal consistency and reasonably high convergent validity, as indicated by the relationship of IHS scores to HQ scores and ULLs. However, IHS scores may also partly reflect the co-occurrence of tinnitus, anxiety, and depression, and this may adversely affect its validity. Two new short questionnaires, the Hyperacusis Impact Questionnaire (HIQ) and the Sound Sensitivity Symptoms Questionnaire (SSSQ), have recently been developed for clinical use. The items on HIQ focus on assessing the impact of hyperacusis on the patient, while the items on SSSQ were designed to assess the type and severity of sound intolerance symptoms based on categories of hyperacusis described by Tyler. Validation of HIQ and SSSQ by Aazh confirmed that these questionnaires can be used in clinical and research settings [42].

As mentioned above, we consider the perception of loudness as the predominant feature in hyperacusis, accompanied by fear and pain. Our aim was to create a tool capturing hyperacusis in these categories and evaluate its psychometric properties.

## 2. Materials and Methods

### 2.1. Setting

Participants were patients admitted to a tertiary referral ENT center in Poland. All participants gave informed consent, and the study was performed in accordance with the Declaration of Helsinki. The study protocol was approved by the local Ethics Committee (KB.IFPS:9/2020).

### 2.2. Hyperacusis Assessment Questionnaire Development

**Figure 1:**
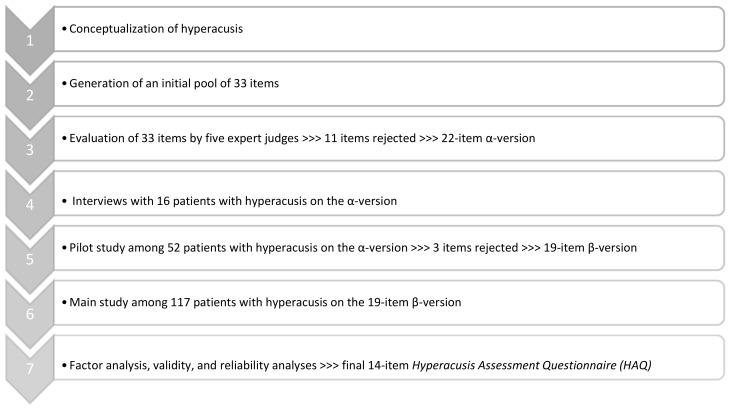
Stages of development of the Hyperacusis Assessment Questionnaire (HAQ).

There were several stages of the new questionnaire development. They are summarized in Figure 1.

Work on the questionnaire began in June 2019. Two experts—an ENT doctor (author DRK) and a psychologist (author EG) analyzed various approaches to decreased sound tolerance in general and hyperacusis in particular. Based on an analysis of the literature and the experience of many years of clinical work, it was concluded that the distinctive feature of hyperacusis is that it appears driven by the subjective, perceived loudness of external sounds. The approach proposed by Tyler et al. [1] was most useful, but there is one exception. In our opinion, annoyance is not a defining characteristic of hyperacusis because it relates to reactions to specific sounds, primarily those produced by humans or repetitive sounds that are unnecessarily generated. Moreover, negative reactions to these sounds are context-specific. It was concluded that annoyance relates more to misophonia than hyperacusis, so it was decided to include in further work only loudness, pain, and fear because they actually occur in patients reporting HA.

Then, the two authors created an initial pool of 33 items. The content validity of the items was evaluated by 5 expert judges (2 ENT doctors and 3 psychologists working with HA patients). The judges were instructed as follows:

*A questionnaire for assessing hyperacusis is under development in our clinic. We are looking for statements that would most adequately capture the nature and symptoms of hyperacusis as well as patients’ complaints. Please rate the extent to which each statement is useful for assessing hyperacusis. Please make a rating on a scale: 0—completely useless, 1—rather useless, 2—rather useful, 3—definitely useful.*

Judges’ evaluations were analyzed, and, as a result, 11 items were removed—those that were rated as completely useless or rather useless by at least one judge.

Then, a version for patients was prepared. Items were scored on a 5-point Likert scale: definitely not useful (0 points), rather not (1 point), neither yes nor no (2 points), rather yes (3 points), and definitely yes (4 points). In the next step, interviews with patients were conducted. Some patients presented to our clinic for hearing loss or tinnitus and, during the visit, it turned out that they were additionally suffering from hyperacusis. There were 16 patients aged between 21 and 60 years (mean age 45.5 years), made up of 13 women and 3 men. The interviews were intended to test the ease of understanding, relevance, acceptability, and feasibility of the questionnaire. After patients had completed the 22-item α-version, they were asked to answer 4 yes/no questions in a brief questionnaire based on the questionnaire just completed. It was found that all interviewed patients reported that the items were comprehensible and that the content of the items was relevant to their problems with hyperacusis. The patients found the items fully acceptable and feasible. The results of the pilot testing were discussed by the expert panel. No changes were made, so the α-version consisting of 22 items was targeted for a pilot study.

In the pilot study, the study group consisted of 52 patients with hyperacusis (28 women, 21 men, and 3 persons who did not provide gender information), aged 19–73 years (mean 49.3 years). The purpose of this study was to identify the best items. Two criteria were applied: the corrected item–total correlation should be at least 0.5, and the item must not show a floor effect. As an example, the item “I use earplugs/earmuffs in situations that may be too loud for me” was excluded because it turned out that 44.2% of the patients answered definitely not. A total of 3 items were removed. After the pilot study, we had a 19-item β-version, and so we started the main study. Our aim was to gather a clinical study group comprised of patients reporting hyperacusis who had been examined by an ENT doctor and who had done audiological tests. Then the COVID-19 pandemic came, and our work slowed down considerably. However, over nearly 2 years, we managed to gather a group of 117 patients with hyperacusis.

### 2.3. Measures

#### 2.3.1. Interview

The interview concerned various aspects of hyperacusis and tinnitus. Patients were asked about the onset and duration of hyperacusis. They were also questioned about what sounds triggered their hyperacusis, if they were afraid of some sounds, and if they avoided particular noisy situations. Patients were asked if noise aggravated their tinnitus and what was the most troublesome problem for them—tinnitus, hyperacusis, or hearing loss.

#### 2.3.2. Audiological Examination

The audiological examination included pure-tone audiometry, impedance audiometry, and measurement of uncomfortable loudness level (ULL). Hearing thresholds were determined for the right and left ears of each patient at frequencies of 0.125, 0.25, 0.5, 1, 2, 4, and 8 kHz (air conduction) and 0.25, 0.5, 1, 2, and 4 kHz (bone conduction). Impedance audiometry involved recording a tympanometric curve and measurement of the stapedial reflex. Normal middle ear function was verified by 226 Hz tympanometry (tympanometric peak pressure between –100 and +100 daPa and peak compensated static acoustic admittance of 0.2–1.0 mmhos) and ipsilateral and contralateral acoustic reflex thresholds (for 0.5–4 kHz tones).

The object of the ULL test was to identify the minimum level of sound that was judged to be uncomfortably loud by the patient. ULL was tested at frequencies of 1, 2, and 4 kHz with a pure-tone stimulus. The tester gradually made the sound louder, and the patient was instructed to press the button or raise their hand as soon as the sound became uncomfortably loud.

#### 2.3.3. Patient-Reported Outcome Measures (PROMs)

Four PROMs were used to evaluate the validity of the new HAQ: Tinnitus Handicap Inventory, Hyperacusis Questionnaire, State–Trait Anxiety Inventory, and Visual Analog Scales.

The Tinnitus Handicap Inventory (THI) comprises 25 items. For each item, the patient can respond with a “yes” (scored 4 points), “sometimes” (2 points), or “no” (0 points). The sum of all items is the total score. The higher the score, the more severe the tinnitus [34]. The Polish version of THI validated by Skarzynski et al. [43] was used in this study.

The Hyperacusis Questionnaire (HQ) evaluates various hyperacusis symptoms [37]. It comprises 14 items, and the answers are rated on a 4-point scale: “no” (0 points), “yes, a little” (1 point), “yes, quite a lot” (2 points), and “yes, a lot” (3 points). The total score is the sum of the 14 items, with higher scores indicating greater hyperacusis.

The State–Trait Anxiety Inventory (STAI) measures anxiety captured as both state and trait [44]. In our study, only the test sheet for trait anxiety was used. It comprised 20 items rated on a 4-point scale from “almost never” to “almost always”. Higher scores indicate greater anxiety. A Polish version of STAI was used [45].

Visual analog scales (VASs) were used to evaluate three aspects of hyperacusis (loudness, pain, and fear) and misophonia. They consisted of four questions: *Are loud sounds uncomfortable for you? Are loud sounds painful for you? Are you afraid of loud sounds? Are human-produced sounds (e.g., eating, breathing, chewing, sniffing) unpleasant for you?* Patients were asked to put a mark between the ends of a 100-mm-long horizontal line. The left end represented “not at all”, and the right end represented “very much”. When analyzing the results, marks were scored from 0 to 100 [46,47]. The higher the score, the higher the hyperacusis (loudness, pain, fear) and the higher the misophonia.

All the above questionnaires were completed by patients once during a visit to the clinic. At the same time, the patients also filled in the 19-item β-version of HAQ. They were also asked to complete the questionnaire again 7–14 days later and return it to the clinic in a stamped addressed envelope. 

### 2.4. Statistical Analysis

The validity of the new Hyperacusis Assessment Questionnaire (HAQ) and its reliability were assessed. Construct validity was established with exploratory factor analysis (EFA). It was conducted with principal component analysis as the extraction method with Oblimin rotation and Kaiser normalization. EFA was conducted both with eigenvalues greater than 1 and with fixed factors, and the clearest solution was chosen. It was assumed that the cumulative explained variance must be greater than 60%. A minimum loading of 0.5 for each item was taken as the threshold, and items should not have cross-loadings above 0.3 [48,49].

Convergent and divergent validity was assessed using Pearson bivariate correlations with the other questionnaires. The HAQ was predicted to positively correlate with Khalfa’s Hyperacusis Questionnaire, and individual subscales were predicted to positively correlate as follows: HAQ Loudness with VAS Loudness, and HAQ Fear with STAI. It was also hypothesized that hyperacusis measured with HAQ would correlate with tinnitus severity but to a weak or, at most, moderate degree. Correlations between HAQ and misophonia measured on the VAS were expected to be only weak. Correlations higher than 0.8 were classified as very strong, between 0.6 and 0.79 as strong, between 0.3 and 0.59 as moderate, and below 0.3 as weak [50].

Additionally, the groups which, in theory, should differ were compared. Here, it was assumed that patients who reported that the most troublesome problem for them was hyperacusis would have higher scores on HAQ than patients who reported they were most bothered by tinnitus, not hyperacusis. A *t*-test for independent groups was used.

Reliability was gauged in terms of internal consistency and reproducibility. Internal consistency, which refers to the homogeneity of items, was assessed by inter-item correlations and Cronbach’s alpha. At least moderate (above 0.40) correlations between items were expected. According to Nunnally and Bernstein [46], Cronbach’s alpha above 0.7 indicates good internal consistency. Reproducibility (the stability of repeated measurements) was evaluated by intraclass correlation (ICC, with a positive rating over 0.70, according to Terwee et al. [51]). Statistical analysis was performed using IBM SPSS Statistics (v.24), and *p* < 0.05 was considered to be statistically significant.

### 2.5. Participants

The study group consisted initially of 117 patients. All of them reported having hyperacusis, but 106 of the 117 also reported having tinnitus. To make the group more homogeneous, the analysis was limited only to people who had both hyperacusis and tinnitus.

The final study group consisted of 106 patients, comprising 51 men and 55 women. They were aged between 19 and 72 years, and the mean age was 45.2 years (*SD* = 12.4).

## 3. Results

### 3.1. Interview Data

The duration of HA in patients ranged from 0.2 to 60 years (*M* = 7.1; *SD* = 9.9). There were 30 patients (28%) who reported that their HA had lasted up to 1 year; 33 (31%) for 1–5 years; 19 (18%) for 5–10 years; and 19 (18%) over 10 years. There were 5 patients who did not answer the question. The onset of HA was sudden in 45 patients (43%), gradual in 55 (52%), and 6 did not answer the question.

The most often reported types of sounds causing discomfort were high-pitched sounds for 51 patients, loud and impulsive sounds (starting abruptly) for 48 patients, everyday sounds (household appliances, dishes clanking, door slamming, etc.) for 27 patients, and low-frequency sounds for 9 patients. Three patients reported that sounds produced by humans (chewing, swallowing, sneezing, etc.) caused them discomfort.

Seventy-six patients (72%) said they feared some sounds, and 73 patients (69%) avoided some noisy situations (e.g., not attending concerts, not going to the cinema).

Sixty-six (62%) patients reported that noise aggravated their tinnitus, 36 patients said that noise did not make their tinnitus worse (34%), and 4 patients did not answer the question. As the most troublesome problem, 72 patients (68%) indicated tinnitus, 24 (23%) hyperacusis, and 6 (6%) hearing loss; 4 patients did not answer the question.

### 3.2. Results of Audiological Evaluation

**Figure 2:**
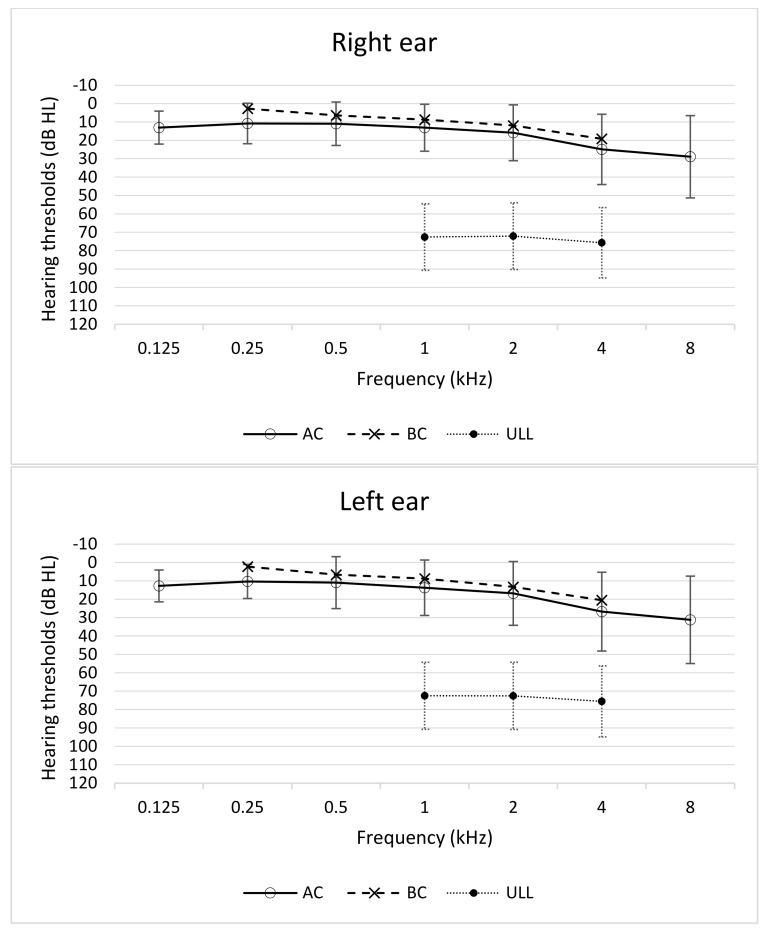
Hearing thresholds (air and bone conduction) and uncomfortable loudness levels. AC, air conduction; BC, bone conduction; RE, right ear; LE, left ear.

The mean hearing thresholds (across all frequencies) were as follows. For air conduction: right ear *M* = 16.8 dB HL (*SD* = 11.8), left ear *M* = 17.7 dB HL (*SD* = 14.1); for bone conduction: right ear *M* = 9.8 dB HL (*SD* = 9.3), left ear *M* = 10.5 dB HL (*SD* = 10.8). Normal tympanometry (tympanogram type A) in both ears was found in 103 patients. Mean uncomfortable loudness levels were as follows. For the right ear: 1 kHz, *M* = 72.6 dB HL (*SD* = 18.1); 2 kHz, *M* = 72.1 dB HL (*SD* = 18.1); 4 kHz: *M* = 75.7 dB HL (*SD* = 19.1). For the left ear: 1 kHz, *M* = 72.5 dB HL (*SD* = 18.3); 2 kHz, *M* = 72.6 dB HL (*SD* = 18.3); 4 kHz, *M* = 75.6 dB HL (*SD* = 19.3). Average hearing thresholds and uncomfortable loudness levels are shown in Figure 2.

### 3.3. Factor Analysis

Exploratory factor analysis (EFA) was initially conducted on all 19 items. The first attempt using eigenvalues greater than 1 resulted in a 4-factor solution (70.4% total variance explained), but the results of the solution were not clear, and the revealed factor structure was difficult to interpret. Then, EFA was conducted again, with a fixed number of factors (3 factors). This time, 64.8% of the total variance was explained, and the factor structure was clear, but with one exception: the item “I feel the best in silence because louder sounds disturb me”. Further attempts were then made to obtain a clear factor structure by removing and reinstating some items.

Finally, the optimal solution was found for the set of 14 items assigned to three factors, which had a reasonable interpretation. The KMO measure of sampling adequacy was 0.86, and Bartlett’s test of sphericity was statistically significant (χ^2^ = 948.87, *p* < 0.001). The three-factor solution explained 70.5% of the total variance. The first factor explained 47.3% of variance (it was subsequently named Loudness Hyperacusis); the second explained 13.9% (named Pain Hyperacusis); and the third 9.3% (Fear Hyperacusis). Table 1 shows factor loadings of the final 14-item Hyperacusis Assessment Questionnaire. Table 1 also shows means and standard deviations for each item, as well as the corrected item–total correlations. The final Hyperacusis Assessment Questionnaire is included in Supplementary Material (see Figures S1 and S2).

### 3.4. Subscales and Global Score of the Hyperacusis Assessment Questionnaire (HAQ)

The HAQ consists of 14 items scored on a 5-point Likert scale: definitely not (0 points), rather not (1 point), neither yes nor no (2 points), rather yes (3 points), definitely yes (4 points).

The subscale scores are calculated by summing up certain answers: for the Loudness Hyperacusis subscale, answers 1, 2, 3, 4, 5, 6, and 7; for the Fear Hyperacusis subscale, 8, 9, 10, and 11; and for the Pain Hyperacusis subscale, 12, 13, and 14. The global score is obtained by summing up the answers from all items. The higher the score, the higher the hyperacusis severity.

**Figure 3:**
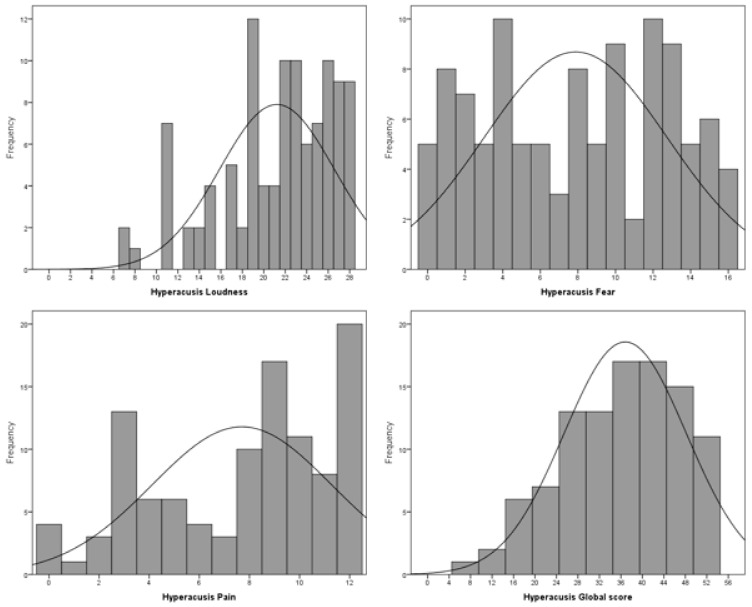
Distributions of the subscales and the global score of HAQ.

Table 2 shows descriptive statistics for the subscales and the global score obtained in the study group. It can be seen that scores were rather high in the Loudness Hyperacusis subscale—the mean score was about 21 points, while the maximum possible score was 28 points. Negative skewness, indicating that most subjects scored in a relatively high range, was present in all subscales as well as in the global score. Small dispersion was apparent in the Loudness Hyperacusis subscale, while in the Fear Hyperacusis and Pain Hyperacusis subscales, outcomes were more varied. This can also be seen in Figure 3, which shows the distribution of subscales and the global score.

Mean and quartiles calculated for the global score could serve as preliminary normative values. The mean value is 36.80 points. The lower quartile, which is 29 points, divides the lowest 25% of the scores, while the upper quartile, which is 45 points, divides the highest 25% of the scores.

Correlations between the subscales were as follows: HAQ Loudness and HAQ Fear, 0.67; HAQ Loudness and HAQ Pain, 0.40; HAQ Fear and HAQ Pain, 0.41. Correlations with HAQ global score were for HAQ Loudness, 0.88; HAQ Fear, 0.87; and HAQ Pain, 0.68.

### 3.5. Relationships between HAQ Scores and Other Measures

Table 3 shows correlations between HAQ and other measures—VASs, HQ, STAI, THI, and ULLs. When it came to correlations with VASs, HAQ Loudness was most strongly correlated with loudness measured on VAS, HAQ Pain was most strongly correlated with pain measured on VAS, and HAQ Fear was most correlated with fear measured on VAS. Correlations between HAQ and misophonia measured on VAS were weak and non-significant.

HAQ subscales and global score were significantly and positively correlated with scores obtained in the Hyperacusis Questionnaire developed by Khalfa et al. [37]. Correlations were moderate; the two highest were between HAQ Loudness and HQ score and between HAQ global score and HQ score.

Trait anxiety measured on STAI was most strongly correlated with HAQ Fear, as expected. Also, tinnitus severity measured on THI was most strongly correlated with HAQ Fear.

Correlations with mean ULLs were statistically non-significant and very weak or weak; the strongest ones were those with HAQ Fear, but they were weak and non-significant. Correlations with average hearing thresholds (air conduction) were very weak and non-significant.

Correlations with age were weak and statistically non-significant: HAQ Loudness, *r* = 0.09, *p* = 0.385; HAQ Pain, *r* = −0.10, *p* = 0.304; HAQ Fear, *r* = 0.07, *p* = 0.497; HAQ global score, *r* = 0.04, *p* = 0.708. Women and men scored similarly on all three subscales and global score; the differences were statistically non-significant. The results were as follows: HAQ Loudness: women *M* = 21.89, *SD* = 5.12; men *M* = 20.51, *SD* = 5.55; *t* = 1.33, *p* = 0.186; HAQ Pain: women *M* = 7.56, *SD* = 3.65; men *M* = 7.84, *SD* = 3.55; *t* = 0.40, *p* = 0.690; HAQ Fear: women *M* = 7.82, *SD* = 4.98; men *M* = 7.94, *SD* = 4.80; *t* = 0.13, *p* = 0.897; HAQ global score: women *M* = 37.27, *SD* = 11.14; men *M* = 36.29, *SD* = 11.73; *t* = 0.44, *p* = 0.661.

Another comparison was made between 72 patients who reported that the most troublesome problem for them was tinnitus and 24 patients who reported they were most bothered by hyperacusis, not tinnitus. Scores obtained in HAQ Loudness, HAQ Pain, HAQ Fear, and HAQ global scores were compared in those two groups, as well as hyperacusis measured on HQ, trait anxiety, and tinnitus severity. As demonstrated in Table 4, those who reported hyperacusis as the most troublesome problem achieved significantly higher scores on HAQ Loudness than those who indicated tinnitus as the most troublesome problem. Also, scores on HAQ Pain, HAQ Fear, and HAQ global score, as well as HQ, were slightly higher in those patients; however, the differences were not statistically significant. The level of trait anxiety was similar in both groups, as well as the level of tinnitus severity.

### 3.6. Internal Consistency and Reproducibility

The internal consistency was high. For HAQ Loudness, α = 0.84; HAQ Fear, α = 0.93; HAQ Pain, α = 0.91; HAQ global, α = 0.91. If item 8 were removed (item belonging to HAQ Loudness), internal consistency would increase to 0.85, so the gain would be negligible. In general, there were no items whose removal would have improved reliability markedly.

The questionnaire was completed again and returned by 101 patients. The time between the two administrations was about 1–2 weeks. In the first administration, means and standard deviations were as follows: HAQ Loudness: *M* = 21.3; *SD* = 5.3; HAQ Fear: *M* = 7.9; *SD* = 4.8; HAQ Pain: *M* = 7.7; *SD* = 3.6; HAQ global: *M* = 36.9; *SD* = 11.2. In the second administration, the results were similar: HAQ Loudness: *M* = 21.4; *SD* = 5.0; HAQ Fear: *M* = 8.0; *SD* = 4.5; HAQ Pain: *M* = 8.1; *SD* = 3.5; HAQ global: *M* = 37.4; *SD* = 10.6.

Intraclass correlation coefficients between the two administrations were as follows: HAQ Loudness, ICC = 0.94 [95% CI, 0.91–0.96]; HAQ Pain, ICC = 0.93 [95% CI, 0.90–0.96]; HAQ Fear, ICC = 0.93 [95% CI, 0.89–0.95]; HAQ global score, ICC = 0.96 [95% CI, 0.93–0.98].

## 4. Discussion

In the clinical practice of our tinnitus department, we often deal with patients who complain of hyperacusis. To date, we have not had a satisfactory tool to objectively measure their complaints. Here, we have aimed to supply clinicians and researchers with a valid and reliable tool to capture hyperacusis in terms of loudness, fear, and pain.

### 4.1. Factor Structure of HAQ

Exploratory factor analysis resulted in a three-factor solution, with 70.5% of the total variance explained. The obtained factor structure was clear and elegant, and all items had loadings above the previously assumed threshold (above 0.5). However, this picture was disturbed by one item: “I avoid situations that could be too loud for me”. This item had a loading of 0.567 on the factor related to Loudness HA but also had a cross-loading of 0.381 on the factor related to Fear HA. We decided not to remove this item because, in our opinion, avoidance behavior related to loud sounds or noisy situations may be driven by fear, and it is difficult to separate loudness and fear in such a case. We decided to include the item in Loudness HA. The structure of the questionnaire provides empirical confirmation of the subcategories of hyperacusis suggested by Tyler et al. [1], except for one subcategory, namely annoyance. In our opinion, annoyance with irritation, anxiety, and tension refers more to misophonia than hyperacusis. The legitimacy of our approach is justified by the very weak correlations with misophonia as measured on VAS.

Also, Greenberg and Carlos [41], the authors of the Inventory of Hyperacusis Symptoms, explicitly refer to Tyler’s concept of hyperacusis and its subcategories. However, their tool contains five factors (general loudness, emotional arousal, psychosocial, functional impact, and communication), and it is not clear how these factors relate to those identified by Tyler. This lack of clarity is highlighted by some questionable solutions, e.g., the item “Sound can cause me pain or psychical discomfort” was included in a dimension called General Loudness, although the item does not mention loudness at all. The authors also admit that most of their 25 items loaded onto one single factor, but 10 items yielded cross-loadings onto two factors (cross-loadings were not shown), and one item onto three factors (and cross-loadings were shown). All this suggests that the factor structure of the tool is not very clear, and it is difficult to say whether it reflects Tyler’s conception of hyperacusis. Such ambiguity is not present in the case of our questionnaire, which plainly confirms that loudness, fear, and pain are the proper categories of hyperacusis.

### 4.2. Correlations with Other Tools Measuring Hyperacusis

In our study, we used the Hyperacusis Questionnaire (HQ) [37], which is the most widely used self-report questionnaire measuring the phenomenon of interest. However, despite its popularity, we still had some concerns about this tool. Although HQ has been used in many studies, it has recently been claimed that it does not provide a valid overall measure of hyperacusis. Fackrell et al. [38], in their in-depth study of its validity and reliability, showed that its psychometric properties are questionable in a tinnitus population. Therefore, we additionally used VASs to assess the convergent validity of our new HAQ. Correlations between subscales of HAQ and HQ were moderate to strong. The lowest was found between Pain HA and HQ score, which is not surprising because HQ does not contain items directly related to pain. When it comes to correlations between HAQ scores and VASs, they were highest between the corresponding dimensions (i.e., Loudness HA was most highly correlated with VAS Loudness, and so on). It should be emphasized that correlations between HAQ scores and misophonia were very weak and statistically non-significant. As mentioned earlier, we decided not to include annoyance as a component of hyperacusis. In our opinion, the key component of annoyance with sounds is a specific reaction to specific sounds. Specificity of reaction is a measure of inappropriateness to the trigger and inadequate emotional saturation (irritation, anger, disgust, anxiety). The specificity of sounds means that they are context-dependent, and their acoustic properties (intensity, frequency, duration) are irrelevant [52]. Based on the Consensus Definition of Misophonia, it is a disorder of decreased tolerance to specific sounds or stimuli associated with such sounds. These stimuli, known as “triggers”, are experienced as unpleasant or distressing and tend to evoke strong negative physiological, emotional, and behavioral responses that are not seen in most other people. Misophonic reactions are not the result of the loudness of auditory stimuli but rather of the specific pattern or meaning assigned by an individual [53].

Jager et al. [54] showed in a large clinical sample of subjects with a confirmed diagnosis of misophonia that for almost all subjects (96%), triggers were eating sounds followed by nasal and breathing sounds (85%); however, visual triggers were also often reported, e.g., repetitive movements (68%). All these considerations mean that, in our opinion, hyperacusis and misophonia should be distinguished.

### 4.3. Hyperacusis and Tinnitus Severity

Schecklman et al. [32] showed in a large sample that 55% (935/1713) of patients with tinnitus also had hyperacusis. Having comorbid hyperacusis was associated with younger age, higher rates of pain disorders, and vertigo. Patients with tinnitus and hyperacusis had significantly higher tinnitus severity, more escalated symptoms of depression, and lower quality of life in comparison with those patients who only had tinnitus. On the other hand, Cederroth et al. [55] found that among 1568 subjects with hyperacusis, 1162 of them (74%) also had tinnitus. And as the severity of hyperacusis increased, the odds ratio for more severe tinnitus also increased. Raj-Koziak et al. [56] revealed that hyperacusis (measured by Khalfa’s Hyperacusis Questionnaire) and tinnitus severity (measured by the Tinnitus Handicap Inventory) were positively correlated (*r* = 0.44). A similar correlation was established by Fackrell et al. [38] (*r* = 0.49). In our study, we found that scores obtained with our new tool were also positively correlated with THI scores, but the relationships were weaker (for Loudness HA, *r* = 0.28; for Fear HA, *r* = 0.36; for global score, *r* = 0.31) or even near zero (for Pain HA, *r* = 0.08). The same is true when it comes to a comparison between hyperacusis measured by the Inventory of Hyperacusis Symptoms (created by Greenberg and Carlos) and tinnitus measured with THI. They found a correlation of 0.58 between IHS and THI (i.e., higher than in our study). Aazh et al. [42] concluded that the Inventory of Hyperacusis Symptoms may partly reflect the co-occurrence of tinnitus. To sum up, the divergent validity of our new tool is good (correlations with tinnitus severity are weak/moderate) and indicates that the measured construct is specifically distinct from tinnitus. Additional confirmation is that patients who reported that the most troublesome problem for them was hyperacusis had higher scores on HAQ than did patients who reported they were most bothered by tinnitus, not hyperacusis.

### 4.4. Hyperacusis and Anxiety

Hyperacusis measured with HAQ was significantly and positively correlated with anxiety (measured on STAI). The strongest correlation was between anxiety and Fear HA (*r* = 0.46); the others were weaker, which was expected. A similar, though stronger, correlation was found by Blaesing and Kroener-Herwig [57]. They used Geräuschberempfindlichkeitsfragebogen (GÜF) [58] to measure distress related to hypersensitivity to sound and STAI to measure anxiety, and the correlation between the scores was 0.65. The researchers found that subjects with hyperacusis and tinnitus showed significantly higher anxiety symptoms than subjects with tinnitus alone and higher than subjects belonging to a control group. Jüris et al. [2] demonstrated that there is an overrepresentation of anxiety disorders and anxiety-related personality traits (e.g., neuroticism) among patients with hyperacusis. Those researchers raised the question of whether there is true causality between anxiety-prone phenotypes and hyperacusis. In our opinion, the term causality may be too strong, and only neuroimaging studies can confirm or reject whether hyperacusis and anxiety share common neural mechanisms.

Ke et al. [59] attempted to determine whether each sound tended to cause a particular subtype of specific reaction (loudness, annoyance, ear pain). The data showed that one patient could have one or more negative reactions to a particular sound. The main triggers of hyperacusis were stress, tension, lack of sleep, fatigue, and being in a large crowd, whereas being in a quiet environment, relaxed or alone, relieved the symptoms.

Fackrell et al. [13] identified the hyperacusis-related problems reported by patients. Twelve domains were grouped into three overarching categories: “Anxiety”, “Reduced quality of life”, and “Physical reaction to sound”. The authors concluded that a patient interview is necessary to explore all potential problems and make an informed decision about hyperacusis treatment.

### 4.5. Hyperacusis, Hearing Thresholds, and Uncomfortable Loudness Level

The mean hearing thresholds for air conduction were about 17 dB HL, and most of the patients had normal hearing. Their ULLs were about 72–76 dB HL on average. The average ULL value for a subject who has no difficulties with sound tolerance is about 100 dB HL [60]. When hyperacusis is present, ULL values are typically in the 65–85 dB HL range [11,30]. ULL values may also be low in cases of misophonia and can be observed in the range of 30 dB HL to 120 dB HL [30]. In our study, correlations between ULLs and HAQ scores were statistically non-significant and weak. Therefore, ULL measurement alone is an insufficient criterion to diagnose hyperacusis or misophonia; it is an auxiliary tool, and hyperacusis is generally diagnosed based on an interview with the patient. This was confirmed in a study in which ULL measurement was found to be neither specific nor sensitive to serve as a single test for hyperacusis [61].

### 4.6. Hyperacusis in Relation to Age and Gender

In our study, we found no relationship between HAQ scores and age, nor with gender. In a scoping review by Ren et al. [8], which included 42 articles, it was concluded that the prevalence of hyperacusis increases with age and that women have a higher prevalence than men. However, in the case of hyperacusis severity, the relationships are not clear-cut. Greenberg and Carlos [41] found that the severity of subjective hyperacusis was rather similar across age groups, and it was similar among men and women. On the other hand, Khalfa et al. [37] demonstrated that women scored higher than men on the Hyperacusis Questionnaire, but they found that, in general, scores did not significantly correlate with age.

### 4.7. Limitations of the Study and Future Directions

The questionnaire was validated in a group of Polish-speaking respondents. The group was representative of Polish patients with hyperacusis and tinnitus because the patients came to our tertiary referral center from all over the country. However, it was just one clinical group consisting of patients with hyperacusis and tinnitus. Our results, therefore, relate to subjects with hyperacusis and comorbid tinnitus, but we do not know whether the same results can be replicated in a group consisting of hyperacusis-affected subjects without tinnitus. Future studies should include a non-clinical sample, serving as a control group. In the future, we would like to cooperate with researchers from different countries to examine whether the revealed three-factor structure of the new tool has cross-cultural validity. In addition, studies on the responsiveness of HAQ are needed if the tool is to be used as an outcome measure for assessing the effectiveness of treatment.

## 5. Conclusions

The present research contributes to the understanding of hyperacusis measurement and increases existing knowledge. Capturing hyperacusis in terms of loudness, fear, and pain seems to be a reasonable approach to assessing this subjective phenomenon.

## Figures and Tables

**Table 1:** Factor loadings of items belonging to the final version of the Hyperacusis Assessment Questionnaire (HAQ).

Item Content	Factor I	Factor II	Factor III	Item Mean (*SD*)	Corrected Item–Total Correlation
1. I perceive louder sounds as annoying	**0.782**	0.077	0.037	3.49 (0.75)	0.615
2. Some sounds, not disturbing to others, are too loud for me	**0.818**	0.108	0.190	3.32 (0.71)	0.511
3. I often find my surroundings too loud, while others are not bothered	**0.700**	−0.042	−0.163	2.86 (1.09)	0.668
4. Some domestic sounds are too loud for me, for example, dishes, cutlery, vacuum cleaner, kitchen mixer, hairdryer	**0.513**	0.005	−0.024	2.67 (1.26)	0.413
5. It is definitely too loud for me at concerts, cinema, or sports events	**0.759**	0.001	−0.013	3.13 (1.12)	0.627
6. I react to loud sounds more strongly than most people	**0.626**	−0.001	−0.271	3.14 (0.99)	0.699
7. I avoid situations that could be too loud for me	**0.567**	−0.154	−0.381	2.80 (1.16)	0.694
8. I often think there will be situations when it will be too loud for me	0.241	−0.053	**−0.772**	2.09 (1.34)	0.776
9. I am afraid of all loud sounds	0.157	0.167	**−0.728**	2.11 (1.31)	0.475
10. I fear that in a while I will hear a loud sound, unpleasant for me	−0.096	0.089	**−0.955**	1.81 (1.35)	0.507
11. I am afraid that I will be exposed to loud sounds	−0.028	0.066	**−0.943**	1.92 (1.36)	0.515
12. Some sounds cause me ear pain and/or headache	0.027	**0.923**	−0.002	2.57 (1.35)	0.822
13. After spending time in noisy places, I have ear pain and/or headache	0.060	**0.861**	−0.025	2.60 (1.21)	0.711
14. Some sounds are so unpleasant that they are painful	−0.026	**0.898**	−0.098	2.56 (1.33)	0.743

**Table 2:** Descriptive statistics of subscale scores and global score for HAQ.

	Min	Max	*M*	*SD*	*Q1*	*Me*	*Q3*	*Skewness*	*Kurtosis*
HAQ Loudness	7	28	21.22	5.35	19	22	26	−0.81	−0.04
HAQ Fear	0	16	7.88	4.87	4	8	12	−0.02	−1.29
HAQ Pain	0	12	7.70	3.59	4	9	11	−0.51	−0.94
HAQ Global score	7	56	36.80	11.39	29	38	45	−0.38	−0.44

Legend: Min, minimum; Max, maximum; *M*, mean, *SD*, standard deviation; *Q1*, lower quartile; *Me*, median; *Q3*, upper quartile.

**Table 3:** Correlations between HAQ scores and other measures.

	VAS L	VAS F	VAS P	VAS M	KHQ	STAI	THI	ULL RE	ULL LE	AC RE	AC LE
*M*; *SD*	67.6; 23.5	61.2; 29.4	42.6; 32.4	36.8; 33.5	21.7; 8.7	47.5; 9.3	56.1; 26.4	73.4; 17.8	73.5; 18.1	16.9; 11.8	17.7; 14.1
HAQ-L	0.62 ***	0.60 ***	0.37 ***	0.16	0.69 ***	0.33 ***	0.28 **	0.05	0.03	0.08	0.11
HAQ-F	0.50 ***	0.67 ***	0.36 ***	0.18	0.56 ***	0.46 ***	0.36 ***	0.19	0.10	0.03	0.04
HAQ-P	0.26 **	0.34 ***	0.77 ***	0.05	0.40 ***	0.11	0.08	−0.09	−0.12	−0.15	−0.13
HAQ global score	0.58 ***	0.69 ***	0.57 ***	0.17	0.69 ***	0.39 ***	0.31 **	0.08	0.02	0.01	0.03

HAQ, Hyperacusis Assessment Questionnaire; VAS, Visual Analog Scale; L, loudness; P, pain; F, fear; M, misophonia; KHQ, Khalfa’s Hyperacusis Questionnaire; STAI, State–Trait Anxiety Inventory; THI, Tinnitus Handicap Inventory; ULL, uncomfortable loudness level; AC, air conduction; RE, right ear; LE, left ear. *** *p* < 0.001; ** *p* < 0.01.

**Table 4:** HAQ scores in patients with tinnitus or hyperacusis as the most troublesome problem.

	Tinnitus as the Most Troublesome Problem (*n* = 72)		Hyperacusis as the Most Troublesome Problem (*n* = 26)		*t*	*p*
	*M*	*SD*	*M*	*SD*		
HAQ Loudness	20.28	5.74	22.83	3.94	2.02	0.046
HAQ Fear	7.35	4.69	8.71	5.29	1.53	0.237
HAQ Pain	7.28	3.68	8.54	3.45	1.48	0.143
HAQ global score	34.90	11.70	40.08	10.41	1.19	0.057
HQ	20.88	8.67	22.88	9.81	0.95	0.346
STAI	47.65	9.17	47.38	11.11	0.12	0.903
THI	58.83	23.70	53.50	33.51	0.79	0.433

## Data Availability

The data presented in this study are available on request from the corresponding author.

## Supplementary Materials

The following supporting information can be downloaded at https://www.mdpi.com/article/10.3390/jcm12206622/s1, Figure S1: Kwestionariusz Nadwrażliwości Słuchowej; Figure S2: Hyperacusis Assessment Questionnaire.
