# Supplementary material for: Hyperacusis Assessment Questionnaire—A New Tool Assessing Hyperacusis in Subjects with Tinnitus
Source: J Clin Med. 2023 Oct 19;12(20):6622. doi: 10.3390/jcm12206622 (PMC10607047; doi:10.3390/jcm12206622)
Supplement: Supplementary file 1 [file jcm-12-06622-s001.zip › jcm-2515232 Suppl 1_Figure S1.pdf]

KWESTIONARIUSZ NADWRAŻLIWOŚCI SŁUCHOWEJ (KNS)

Nazwisko:		Imię:
Wiek:	Płeć: M / K	Data:

Poniżej znajdują się stwierdzenia dotyczące tego, jak ludzie mogą odbierać i reagować na różne dźwięki. Proszę uważnie przeczytać każde z tych stwierdzeń i zastanowić się, czy opisują one Pana/Pani sytuację.

Proszę o zaznaczenie jednej z podanych odpowiedzi. Poszczególne cyfry oznaczają:

0 – zdecydowanie nie 1 – raczej nie 2 – ani tak, ani nie 3 – raczej tak 4 – zdecydowanie tak

1.	Głośniejsze dźwięki odbieram jako dokuczliwe.	0	1	2	3	4
2.	Niektóre dźwięki, które innym nie przeszkadzają, są dla mnie za głośne.	0	1	2	3	4
3.	Często uważam, że wokół mnie jest za głośno, podczas gdy innym to nie przeszkadza.	0	1	2	3	4
4.	Niektóre dźwięki w gospodarstwie domowym są dla mnie za głośne, np. wydawane przez talerze, sztućce, odkurzacz, mikser, suszarkę do włosów.	0	1	2	3	4
5.	Jest dla mnie zdecydowanie za głośno na koncertach, w kinie, podczas imprez sportowych.	0	1	2	3	4
6.	Silniej niż większość ludzi reaguję na głośne dźwięki.	0	1	2	3	4
7.	Unikam sytuacji, w których mogłoby być dla mnie za głośno.	0	1	2	3	4
8.	Często myślę o tym, że będą takie sytuacje, w których będzie dla mnie za głośno.	0	1	2	3	4
9.	Obawiam się wszystkich głośnych dźwięków.	0	1	2	3	4
10.	Towarzyszy mi uczucie lęku, że za chwilę usłyszę głośny, nieprzyjemny dla mnie dźwięk.	0	1	2	3	4
11.	Boję się, że będę narażony na głośne dźwięki.	0	1	2	3	4
12.	Niektóre dźwięki powodują u mnie ból uszu i/lub głowy.	0	1	2	3	4
13.	Po przebywaniu w hałaśliwych miejscach odczuwam ból w uszach i/lub głowie.	0	1	2	3	4
14.	Niektóre dźwięki są dla mnie tak nieprzyjemne, że aż bolesne.	0	1	2	3	4
