# Supplementary material for: Hyperacusis Assessment Questionnaire—A New Tool Assessing Hyperacusis in Subjects with Tinnitus
Source: J Clin Med. 2023 Oct 19;12(20):6622. doi: 10.3390/jcm12206622 (PMC10607047; doi:10.3390/jcm12206622)
Supplement: Supplementary file 1 [file jcm-12-06622-s001.zip › jcm-2515232 Suppl 2_Figure S2.PDF]

WORLD HEARING CENTER
OF THE INSTITUTE OF PHYSIOLOGY AND PATHOLOGY OF HEARING

HYPERACUSIS ASSESSMENT QUESTIONNAIRE (HAQ)

Surname:		Name:
Age:	Sex: M / F	Date:

Below are statements about how people may perceive and react to different sounds. Please, read each statement carefully and consider whether it describes your situation.

Please mark one of the provided answers. The individual numbers mean:

0 – definitely not   1 – rather not   2 – neither yes nor no   3 – rather yes   4 – definitely yes

1.	I perceive louder sounds as annoying.	0	1	2	3	4
2.	Some sounds, not disturbing to others, are too loud for me.	0	1	2	3	4
3.	I often find my surroundings too loud, while others are not bothered.	0	1	2	3	4
4.	Some domestic sounds are too loud for me, for example dishes, cutlery, vacuum cleaner, kitchen mixer, hairdryer.	0	1	2	3	4
5.	It is definitely too loud for me at concerts, cinema, or sports events.	0	1	2	3	4
6.	I react to loud sounds more strongly than most people.	0	1	2	3	4
7.	I avoid situations that could be too loud for me.	0	1	2	3	4
8.	I often think there will be situations when it will be too loud for me.	0	1	2	3	4
9.	I am afraid of all loud sounds.	0	1	2	3	4
10.	I fear that in a while I will hear loud sound, unpleasant for me.	0	1	2	3	4
11.	I am afraid that I will be exposed to loud sounds.	0	1	2	3	4
12.	Some sounds cause me ear pain and/or headache.	0	1	2	3	4
13.	After spending time in noisy places, I have ear pain and/or headache.	0	1	2	3	4
14.	Some sounds are so unpleasant for me, that they are painful.	0	1	2	3	4
